# Transepithelial Transport and Enzymatic Detoxification of Gluten in Gluten-Sensitive Rhesus Macaques

**DOI:** 10.1371/journal.pone.0001857

**Published:** 2008-03-26

**Authors:** Michael T. Bethune, Erin Ribka, Chaitan Khosla, Karol Sestak

**Affiliations:** 1 Tulane National Primate Research Center, Covington, Louisiana, United States of America; 2 Department of Biochemistry, Stanford University, Stanford, California, United States of America; 3 Department of Chemistry, Stanford University, Stanford, California, United States of America; 4 Department of Chemical Engineering, Stanford University, Stanford, California, United States of America; 5 Department of Microbiology and Immunology, Tulane University School of Medicine, New Orleans, Louisiana, United States of America; Instituto Oswaldo Cruz and FIOCRUZ, Brazil

## Abstract

**Background and Aims:**

In a previous report, we characterized a condition of gluten sensitivity in juvenile rhesus macaques that is similar in many respects to the human condition of gluten sensitivity, celiac disease. This animal model of gluten sensitivity may therefore be useful toward studying both the pathogenesis and the treatment of celiac disease. Here, we perform two pilot experiments to demonstrate the potential utility of this model for studying intestinal permeability toward an immunotoxic gluten peptide and pharmacological detoxification of gluten *in vivo* by an oral enzyme drug candidate.

**Methods:**

Intestinal permeability was investigated in age-matched gluten-sensitive and control macaques by using mass spectrometry to detect and quantify an orally dosed, isotope labeled 33-mer gluten peptide delivered across the intestinal epithelium to the plasma. The protective effect of a therapeutically promising oral protease, EP-B2, was evaluated in a gluten-sensitive macaque by administering a daily gluten challenge with or without EP-B2 supplementation. ELISA-based antibody assays and blinded clinical evaluations of this macaque and of an age-matched control were conducted to assess responses to gluten.

**Results:**

Labeled 33-mer peptide was detected in the plasma of a gluten-sensitive macaque, both in remission and during active disease, but not in the plasma of healthy controls. Administration of EP-B2, but not vehicle, prevented clinical relapse in response to a dietary gluten challenge. Unexpectedly, a marked increase in anti-gliadin (IgG and IgA) and anti-transglutaminase (IgG) antibodies was observed during the EP-B2 treatment phase.

**Conclusions:**

Gluten-sensitive rhesus macaques may be an attractive resource for investigating important aspects of celiac disease, including enhanced intestinal permeability and pharmacology of oral enzyme drug candidates. Orally dosed EP-B2 exerts a protective effect against ingested gluten. Limited data suggest that enhanced permeability of short gluten peptides generated by gastrically active glutenases may trigger an elevated antibody response, but that these antibodies are not necessarily causative of clinical illness.

## Introduction

Celiac disease is an inheritable enteropathy caused by dietary gluten from common food grains such as wheat, rye, and barley [1]. Unlike most other dietary proteins, gluten is incompletely digested by gastrointestinal proteases, yielding proteolytically resistant peptides that trigger a deleterious immune response in genetically susceptible individuals [2]. To widely varying degrees in celiac patients, this immune response produces intestinal effects, such as mucosal damage, malabsorption, and clinical diarrhea, as well as systemic humoral effects, such as the production of anti-gliadin antibodies (AGA) and anti-transglutaminase 2 (TG2) autoantibodies [3], [4]. The only existing therapy for celiac disease is a gluten-free diet, which is effective in most celiac patients when strictly followed [5]. However, the maintenance of this diet is extremely difficult due to the ubiquity of gluten in most human diets, and relapse due to accidental ingestion of gluten contained in inadequately labeled or gluten-contaminated foods is an abiding concern and a frequent occurrence for treated celiac patients [6]–[9].

Several properties of immunogenic gluten peptides have been identified as being critical toward their pathogenic role in celiac disease [10]. These include proteolytic resistance due to high proline and glutamine content [2], [11], presence of preferred glutamine residues for TG2-mediated glutamine deamidation [12], [13], and high affinity of these deamidated sequences for human leukocyte antigen (HLA)-DQ2 [14], [15], a class II major histocompatibility complex (MHC) molecule associated with over 90% of diagnosed celiac patients [16], [17]. Additionally, longer peptides containing multiple epitopes appear to be more immunotoxic than their constituent epitopes [2], [18]. One peptide in particular, a 33-mer derived from *in vitro* gastrointestinal proteolysis of α2-gliadin, possesses all of these characteristics and activates proliferation in all celiac patient-derived T cell lines tested so far [2], [19]. Moreover, this peptide is detected as a stable digestive product of ingested gluten in both rats [20] and cynomolgus monkeys (MTB, unpublished results), suggesting it is likely produced from dietary gluten in the human gut as well. It is therefore presumed that the 33-mer is important in the induction and propagation of the adverse response to gluten in celiac disease patients. To enact such a role *in vivo*, however, this relatively large (∼4 kDa) peptide must be transported intact across the mucosal epithelium to the underlying lymphoid tissue where it can be presented by DQ2 on antigen presenting cells to gluten-specific CD4+ T cells.

Despite strong evidence implicating gluten peptides in celiac disease pathogenesis, the detection of a chemically-defined gluten peptide in extra-intestinal body fluids following oral dosing has yet to be reported, and the extent to which disease state alters permeability toward such a peptide is unclear. It is well established that active enteropathy is associated with structurally altered tight junctions [21] and increased permeability toward small (<1 kDa) non-permeating molecules [22]–[26], and that these defects are not completely resolved by a gluten-free diet [21], [23]–[25]. However, it is unclear whether these findings will translate to increased permeability toward gluten peptides of sufficient size to elicit an immune response. Further, studies of intestinal permeability with respect to disease state in celiac patients are hampered by the practical difficulty of ensuring strict dietary exclusion of gluten [6]–[9]. An animal model of gluten sensitivity could enable the observation and study of transepithelial gluten peptide delivery in an *in vivo* system in which dietary gluten content can be absolutely controlled. Furthermore, such an animal model could facilitate preclinical testing of pharmacological therapies aimed at protecting celiac patients from the constant risk of dietary gluten exposure.

In a previous report, we characterized a condition of gluten sensitivity in juvenile rhesus macaques (*Macaca mulatta*) that is similar in many respects to celiac disease [27]. Affected animals exhibited intestinal lesions, malabsorptive steatorrhea, clinical diarrhea, and elevated anti-gliadin (though not anti-TG2) antibodies in response to a gluten-containing diet. Administration of a gluten-free diet to one such affected animal, FH09, caused complete remission, whereas reintroduction of dietary gluten caused clinical, histological, and serological relapse. Similar dietary changes had no effect on an age-matched control, FR26.

In this report, we conduct two pilot studies using the gluten-sensitive macaque model. The first study investigates the fundamental question of whether the immunotoxic 33-mer peptide is delivered intact across the intestinal epithelium in gluten-sensitive FH09, during both remission and relapse, as well as in healthy control FR26. We use mass spectrometry to detect for the first time the transepithelial delivery of a chemically-defined, immunotoxic gluten peptide in a gluten-sensitive organism. The second study investigates the practical question of whether oral protease therapy can protect a second gluten-sensitive animal, FH45, from gluten challenge-induced relapse. We present clinical and serological data for FH45, and for control FI96, showing that EP-B2, a gluten-specific endoprotease with potential as a therapeutic for celiac disease [20], [28]–[30], prevents clinical relapse in FH45 in response to a gluten challenge. However, this treatment gives rise to an unexpected elevation in anti-gliadin and anti-TG2 antibodies, the implications of which are discussed.

## Methods

### Rhesus macaques

Gluten-sensitive juvenile macaques, FH09 and FH45, were selected for dietary treatment and pilot experiments from a population of rhesus macaques exhibiting clinical diarrhea and elevated AGA on a gluten-containing diet [27]. Age-matched controls, FR26 and FI96, were selected from a panel of AGA-negative, healthy rhesus macaques tested in the same study. Animals were housed for 10 months under biosafety level two conditions in accordance with the standards of the Association for Assessment and Accreditation of Laboratory Animal Care. Investigators adhered to the Guide for the Care and Use of Laboratory Animals prepared by the National Research Council.

### Gluten-containing and gluten-free diets

The gluten-containing and gluten-free diets used in this study were previously described [27]. Animals would typically consume 4% of their body weight daily (e.g. 160 g of food for a 4 kg animal).

*Veterinary procedures: 33-mer intragastric inoculation, EP-B2 administration, peripheral blood collection, and clinical evaluation.*

#### Study 1

Gluten-sensitive FH09 and control FR26 were administered a gluten-free diet until FH09 was negative for AGA and in clinical remission [27]. A dose of 25 mg (6.5 µmol) of isotopically labeled 33-mer (^D3^33-mer) dissolved in 10 ml of Gatorade was administered directly into the stomach of FH09 by intragastric tube. A 0.5 ml sample of EDTA-blood was collected from an ear vein at 0, 30, 60, 120, and 180 min following the 33-mer inoculation. Dietary gluten was then reintroduced for 10 weeks until FH09 was AGA-positive and in clinical relapse [27]. A dose of 25 mg of ^D9^33-mer was administered exactly as described above to FH09 and FR26, and EDTA-blood was collected from an ear vein at 0, 60, 120, 180, and 240 min following 33-mer inoculation. Animals were fasted at least 2 h prior to peptide inoculations. Blood collections and 33-mer inoculations were performed with sedated and anesthetized animals.

#### Study 2

Gluten-sensitive FH45 and control FI96 were switched from a gluten-containing to a gluten-free diet at week 0, re-challenged with the gluten-containing diet from week 10.4 to 11.4, and then returned to a gluten-free diet for the remainder of the experiment. To test whether orally administered EP-B2 can prevent clinical symptoms resulting from gluten challenge, animals were fed a gluten-free diet with the addition of a daily slice of unbleached wheat bread (∼4 g gluten) for 8 weeks (weeks 22.9 to 30.9), during the first 4 of which (weeks 22.9 to 26.9) gluten-sensitive macaque FH45 received a daily dose of 2 g recombinant proEP-B2 dissolved in an electrolyte drink immediately prior to bread consumption. Recombinant proEP-B2 was expressed and purified as previously described [28], [31]. Peripheral blood samples (1 ml) were collected every 1–2 weeks from a femoral vein, and plasma was harvested and stored at −80^o^C until analyzed for AGA and anti-TG2 antibodies. Clinical health was monitored as previously described [27]. Briefly, daily clinical scores were averaged over weekly intervals to obtain each weekly mean±standard deviation. Criteria for clinical scoring (1–6 scale) were scaled relative to “clinically normal”, age-matched controls, i.e. score of 1. Score 2 corresponded to beginning of diarrhea, i.e. pasty stools. Score 3 corresponded to semi-liquid stools and decreased activity. Score 4 corresponded to liquid stools, decreased activity, moderate dehydration and “balloon” stomach. Score 5 corresponded to liquid stools, depression, severe dehydration and balloon stomach. Score 6 corresponded to a moribund animal where prompt euthanasia is recommended.

### Indirect ELISA for AGA and anti-TG2 antibodies

ELISA assays were performed as previously described [27].

### Peptide detection via 3Q LC-MS/MS

Unlabeled and isotopically labeled 33-mer peptides (LQLQPFPQPQLPYPQPQLPYPQPQLPYPQPQPF; positions 3,11, and 25 underlined) were synthesized on solid-phase using Boc/HBTU chemistry, purified post-cleavage by reverse-phase HPLC, and lyophilized as previously described [18]. Deuterium labels were introduced by incorporating [5,5,5-D_3_]-leucine (Cambridge Isotope Laboratories, Inc.) at position 11 (^D3^33-mer), or at positions 3, 11, and 25 (^D9^33-mer) during synthesis. The identity of each purified peptide was confirmed by electrospray mass spectrometry prior to lyophilization. Following peptide dosing and peripheral plasma collection, described above, plasma proteins were precipitated by addition of acetonitrile. Plasma was mixed with an equal volume of cold acetonitrile+0.1% formic acid, vortexed, incubated for 2 h at 4°C, and centrifuged at 14,000•g for 10 min at 4°C. The supernatant was mixed with an equal volume of 0.1% formic acid to dilute the acetonitrile concentration, and 100 µl of each supernatant was injected on 3Q LC-MS/MS. When quantitation was performed, unlabeled 33-mer was spiked into the plasma samples prior to acetonitrile precipitation as an internal control (5 pmol/100 µl injection) and injections were performed at least in triplicate.

Mass spectrometry analysis was performed as previously described [20] with the following modifications. For ^D3^33-mer detection, positive ion SRM mode was used for monitoring the transitions of ions at *m/z* 979.5^4+^→263.4^+^ (30V cone voltage, 27eV collision energy) and *m/z* 1305.6^3+^→263.1^+^ (40V cone voltage, 50eV collision energy). For ^D9^33-mer, the transitions monitored were *m/z* 981.0^4+^→263.1^+^ (30V cone voltage, 27eV collision energy.) for the quantitation assay and *m/z* 1307.7^3+^→263.1^+^ (40V cone voltage, 50eV collision energy) as a confirmatory transition. For unlabeled 33-mer internal standard, the transitions monitored were *m/z* 978.8^4+^→263.1^+^ (30V cone voltage, 27eV collision energy) for the quantitation assay and *m/z* 1304.7^3+^→263.1^+^ (40V cone voltage, 50eV collision energy) as a confirmatory transition. It was confirmed that plasma samples yielded no signal from unlabeled 33-mer transitions absent internal standard spiking. Levels of ^D9^33-mer in each sample were determined by comparison of the area under the ^D9^33-mer transition peak to the area under the internal standard transition peak. To enable statistical evaluation in cases where the ^D9^33-mer peak area was below threshold but the internal standard was detected, below-threshold ^D9^33-mer peak areas were assigned the threshold value. In all such instances, this resulted in a narrowing of the difference between FH09 samples and time-matched FR26 controls.

### Statistical evaluation

Statistical differences between the level of plasma ^D9^33-mer in FH09 and FR26 at matched timepoints following oral dosing were determined by Student's t-test assuming equal variances. A p value of <0.05 was considered significant.

## Results

### Study 1: Intestinal permeability toward orally dosed 33-mer

#### Detection of ingested gluten peptide in the plasma of a gluten-sensitive macaque in remission

**Figure 1:**
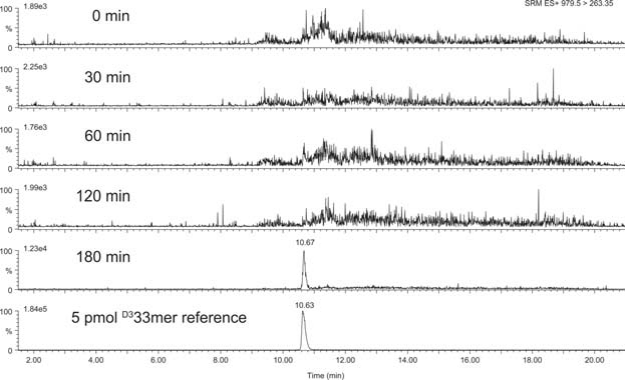
Detection of an oral gluten peptide in the peripheral plasma of gluten-sensitive rhesus macaque FH09 during remission. Plasma samples collected at indicated timepoints following intragastric administration of ^D3^33-mer to FH09 (in remission) were analyzed by 3Q LC-MS/MS, monitoring for the *m/z* 979.5^4+^→263.4^+^ transition in the positive ion SRM mode. Peak intensities are scaled relative to the highest peak in each chromatogram. Absolute intensities are indicated to the right of the ordinate axis. A ^D3^33-mer peptide reference exhibits the same retention time and ion transitions as the peptide detected in the 180 min plasma sample.

Gluten-dependent enteropathy in gluten-sensitive macaques and celiac patients does not necessarily require absorption of gluten peptides across the epithelium, since gluten peptides have been shown to induce epithelial damage directly via the innate immune pathway [32]. However, the adaptive humoral immune response raised against gluten in both macaques and humans suggests that gluten peptides are delivered intact across the intestinal epithelium, thus gaining access to the underlying lymphoid tissue. Despite this, the detection of an intact, chemically-defined gluten peptide in an extra-intestinal compartment following oral ingestion has yet to be reported. To address this, we orally dosed a synthetic, isotopically labeled gluten peptide to gluten-sensitive macaque FH09 in remission, and measured peripheral plasma peptide levels over time via mass spectrometry. A dose of 25 mg (6.5 µmol) of triply deuterated 33-mer (^D3^33-mer) dissolved in 10 ml of electrolyte drink was administered intragastrically to FH09, and plasma samples were collected from an ear vein at 0, 30, 60, 120, and 180 min following gavage. Following acetonitrile precipitation of plasma proteins, ^D3^33-mer content was assessed by selected reaction monitoring (SRM) analysis using high-performance liquid chromatography-coupled triple quadrupole tandem mass spectrometry (3Q LC-MS/MS). The peptide was detected in plasma collected at 180 minutes after oral dosing, but was not detected in earlier post-dose samples, nor in plasma collected prior to dosing (Figure 1). By comparison to a 5 pmol standard injected in parallel, the isotopically labeled peptide was estimated to be at nanomolar concentrations in the plasma. Since SRM analysis requires that selected precursor ions, as well as expected fragment ions, be detected in order to constitute a transition signal, the detection of ^D3^33-mer signal in a plasma sample following oral dosing demonstrates that ingested ^D3^33-mer was delivered intact across the intestinal epithelium. As the peptide was dosed to FH09 following adherence to a gluten-free diet, at which point the mucosa had ostensibly recovered [27], this finding implies that the gluten-sensitive macaque gut remains permeable to large (∼4 kDa) gluten peptides even in a state of remission.

#### 33-mer is absorbed by a gluten-sensitive macaque but not by a healthy control

**Figure 2:**
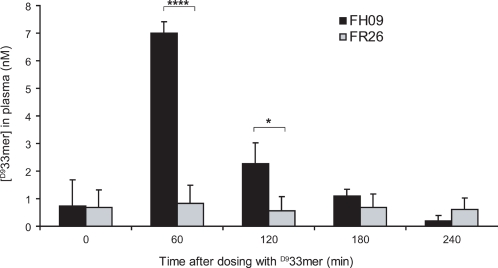
Intact absorption of an oral gluten peptide in gluten-sensitive rhesus macaque FH09 but not in healthy control FR26. Plasma samples collected at indicated timepoints following intragastric administration of ^D9^33-mer to FH09 (with active enteropathy) and FR26 were analyzed by 3Q LC-MS/MS. Plasma concentrations of ^D9^33-mer were determined by comparison to an unlabeled 33-mer internal standard. Mean±SD displayed from at least 3 injections. *P<0.05, ****P<0.00005.

To determine how disease state affects the transepithelial delivery of intact gluten peptides, we induced clinical relapse in FH09 by reintroduction of dietary gluten for 10 weeks [27], then performed a second peptide dosing experiment with FH09 in a state of active enteropathy and with FR26 as a healthy control. In this experiment, we used ^D9^33-mer, rather than ^D3^33-mer, to provide a large enough difference between the mass of our test peptide and that of an unlabeled 33-mer internal standard, thereby enabling quantitation. Both FH09 and FR26 were dosed with 25 mg of isotopically labeled peptide (^D9^33-mer), and peripheral plasma samples were collected as before at 0, 60, 120, 180, and 240 minutes. Serosal ^D9^33-mer peptide was detected at 60 minutes, and possibly at 120 minutes, following oral dosing in FH09, but was not detected above background levels at any timepoint in the healthy control FR26 (Figure 2). By comparison to the internal standard, we calculated the concentration of ^D9^33-mer in peripheral plasma at 60 minutes to be 7.0±0.4 nM. Since FH09 weighed 2.58 kg at this point, and therefore had approximately 260 ml of peripheral blood, we estimate that <0.1% of the 6.5 µmol ingested peptide was delivered intact across the intestinal epithelium.

### Study 2: Pharmacological protection from gluten challenge by oral enzyme therapy

#### Oral glutenase therapy can prevent clinical relapse in response to dietary gluten

Orally administered proteases capable of digesting immunotoxic gluten peptides constitute a promising potential therapy for celiac disease [33], [34]. Recently, barley endoprotease EP-B2 was shown to destroy immunotoxic gluten epitopes, including those present in the 33-mer [28], thereby extirpating gluten's immunogenicity toward celiac patient-derived, gluten-reactive T cells [29]. Due to the lack of an animal model for celiac disease, evaluation of enzyme efficacy *in vivo* has been limited to experiments performed in rats, which are not gluten-sensitive, requiring that digested material be removed from the alimentary tract and tested for immunogenicity *ex vivo* [20]. This approach cannot therefore address a central consideration of oral protease therapy regarding the kinetics of deleterious responses to ingested gluten versus the kinetics of glutenase-catalyzed detoxification. Preclinical demonstration of EP-B2 efficacy in a gluten-sensitive organism would benefit the evaluation of this enzyme as a potential drug candidate.

**Figure 3:**
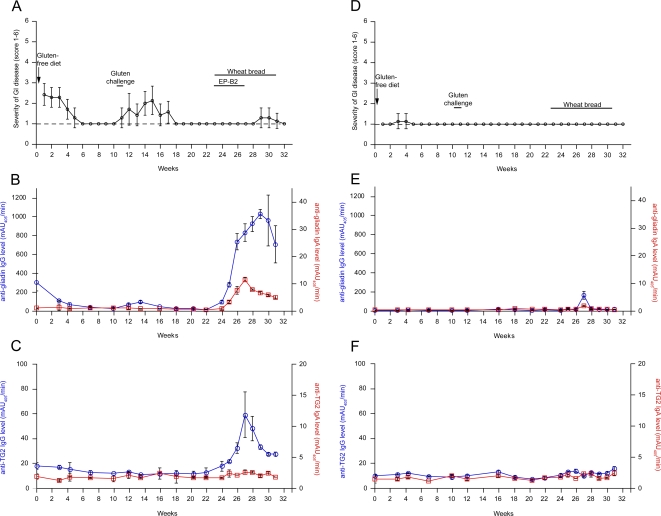
Oral enzyme therapy prevents clinical relapse but increases plasma AGA and anti-TG2 IgG in gluten-sensitive rhesus macaque FH45. Gluten-sensitive FH45 (A-C) and control FI96 (D-F) were switched from a gluten-containing to a gluten-free diet at week 0, re-challenged with the gluten-containing diet from week 10.4 to 11.4, and then returned to a gluten-free diet for the remainder of the experiment. During week 22.9-30.9, the gluten-free diet was supplemented with a daily slice of wheat bread. FH45 additionally received a daily dose of EP-B2 glutenase during the first half of this period (week 22.9-26.9). Clinical scores (A,D), anti-gliadin antibodies (B,E), and anti-TG2 antibodies (C,F) were monitored over the course of the experiment. Levels of IgG (blue; open circles) and IgA (red; open squares) antibodies are provided on the left and right ordinates, respectively. Clinical score criteria are described in Methods.

To identify a gluten-sensitive macaque for this study, and to corroborate the gluten-dependence of clinical and serological signs and symptoms observed in FH09 [27], we administered a gluten-free diet to FH45, an AGA+ macaque with chronic diarrhea of lesser severity compared with FH09, as well as to FI96, an age-matched control macaque without AGA or clinical symptoms in response to gluten. Similar to FH09, the administration of a gluten-free diet resulted in clinical remission in FH45 within 6 weeks, over which time AGA levels also returned to baseline values (Figure 3). Upon transient re-introduction of gluten (week 10.4–11.4), FH45 relapsed clinically and exhibited low levels of AGA, both of which recovered once again with return to a gluten-free diet (Figure 3A and B). As with previous control FR26 [27], control FI96 exhibited no clear clinical responses to these dietary changes (Figure 3D).

To determine whether oral enzyme therapy can prevent clinical responses to a dietary gluten challenge in an otherwise gluten-free diet, we initiated our pilot study with gluten-sensitive FH45 in a state of clinical remission (week 22.9). At this point, we supplemented the gluten-free diet fed to macaques FH45 and FI96 with a daily dose of gluten (slice of wheat bread) for 8 weeks, during the first 4 of which FH45 additionally received a daily dose of EP-B2 immediately preceding bread consumption. Clinical health and antibody levels were monitored during this period (Figure 3). Importantly, FH45 remained clinically healthy while being administered EP-B2 over the first 4 weeks of this gluten challenge, even though previous gluten challenges in FH45 and FH09 resulted in rapid (1–2 weeks) relapse (Figure 3A and [27]). Indeed, FH45 displayed signs of clinical illness ∼1 week after EP-B2 dosing was concluded, and continued to exhibit sporadic diarrhea over the remainder of the gluten challenge. By contrast, FI96 did not respond clinically to wheat bread supplementation (Figure 3D).

#### Oral glutenase therapy causes increased production of AGA and anti-TG2 antibodies

Although we expected AGA in FH45 to remain low during EP-B2 treatment, we instead observed a striking inverse correlation between clinical score and AGA levels, with AGA increasing during EP-B2 treatment, and declining thereafter, even while wheat bread dosing continued. The gluten dose from wheat bread supplementation was ∼10% of that received from the gluten-containing diet, yet AGA levels were substantially higher (∼3-fold) in FH45 duing wheat bread/EP-B2 administration than they were in response to the gluten-containing diet fed prior to our experiments (Figure 3B, week 0). Even more remarkably, anti-TG2 IgG levels were also elevated in FH45 over the course of EP-B2 treatment (Figure 3C), though these were not observed in gluten-sensitive macaques on a gluten-containing diet absent EP-B2 treatment (Figure 3C and [27]). Like AGA, the level of anti-TG2 IgG correlated inversely with clinical score, declining once EP-B2 treatment ended and diarrhea ensued due to continued gluten challenge. The implications of these unexpected findings are discussed below.

## Discussion

We exploited the gluten-sensitive rhesus macaque model to study two questions regarding gluten sensitive enteropathy that have not yet been investigated in humans or any other model organism. First, can appreciable quantities of immunotoxic gluten peptides be absorbed intact across the enterocyte barrier in gluten-sensitive animals? And second, can oral glutenases provide clinical benefit to these animals upon gluten exposure?

To investigate gastrointestinal absorption of intact gluten peptides, we dosed a gluten-sensitive macaque with an isotopically labeled form of the immunotoxic 33-mer gluten peptide [13], and measured its plasma concentration using a sensitive and specific mass spectrometric method. Nanomolar concentrations of the peptide were measured in peripheral blood, both in remission (gluten-free diet) as well as in active disease (gluten challenge), but not in control animals. Although the concentration of the 33-mer peptide in the intestinal mucosa is likely to be higher, low nanomolar concentrations of the 33-mer peptide are sufficient to stimulate proliferation of celiac patient-derived T cells in culture [2], [19]. Thus, gluten-sensitive macaques appear to exhibit enhanced intestinal permeability akin to celiac disease patients. If so, they may offer a unique opportunity to investigate the mechanisms underlying transport of immunotoxic gluten peptides across the enterocyte barrier, as well as the relevance of this aspect of celiac disease to overall disease pathogenesis.

We also took advantage of these gluten-sensitive macaques to evaluate the clinical and serological efficacy of a therapeutically promising oral glutenase. The gluten detoxifying characteristics of the zymogen form of barley endoprotease EP-B2 have been extensively investigated as a stand-alone drug candidate [20], [28] and in combination with complementary glutenases [29], [30]. Our study revealed that clinically achievable oral doses of the EP-B2 proenzyme, but not placebo, could prevent dietary gluten from precipitating clinical relapse in a gluten-sensitive macaque. Remarkably, however, the levels of anti-gliadin antibodies (IgG and IgA) underwent a dramatic increase, and, for the first time, we observed an anti-TG2 antibody (IgG only) response to dietary gluten in macaques. We speculate that the spike in anti-gliadin antibody levels is due to delivery of a high dose of short gluten peptides into a permeable duodenum upon gastric emptying. Although these shorter peptides are expected to exhibit diminished T cell reactivity, their small size enables them to penetrate the enterocyte barrier more efficiently than the considerably longer peptides produced in the absence of EP-B2. In turn, systemic distribution of these absorbed peptides could elicit an anti-TG2 IgG response because even short gluten peptides (e.g. pentamers) are good substrates of mammalian TG2 [35]–[37]. If so, our findings have two important implications. First, if the goal of oral glutenase therapy is to protect a celiac disease patient from all gluten responses, including anti-gliadin and anti-TG2 antibodies, then gluten must be extensively proteolyzed in the stomach, not simply rendered non-reactive towards disease-specific Th1 cells. The use of combination enzyme therapies that cleave gluten into very short peptides may be beneficial in this regard [29], [30]. Regardless, our data suggest that careful monitoring of patient antibody levels is warranted in future clinical trials for glutenase therapies. Second, there has been considerable debate over the role of anti-TG2 antibodies in celiac disease pathogenesis. For example, celiac IgG and IgA antibodies inhibit TG2 activity *in situ* [38], and subepithelial deposition of IgA class antibodies is targeted against TG2 *in vivo* [39], suggesting that anti-TG2 antibodies may impede the physiological function of TG2. On the other hand, the existence of IgA-deficient celiac patients, as well as the apparent normal intestinal development in TG2 knockout mice [40], argues instead that these antibodies may not be causative of disease. Although we were unable to detect anti-TG2 IgA (suggesting the autoantibodies observed in FH45 were from an extra-intestinal source), our data appears to support the latter hypothesis because the clinical symptoms of this gluten-sensitive macaque correlated inversely with antibody levels.
